# Assessment of emergency medical service of three schemes under the concept “medical emergency care for everybody everywhere”

**DOI:** 10.1186/1471-2458-14-S1-O31

**Published:** 2014-01-29

**Authors:** Orawan Prasitsiriphon, Paibul Suriyawongpaisal, Samrit Srithamrongsawats

**Affiliations:** 1 Health Insurance System Research Office, Nonthaburi 11000, Thailand; 2 Faculty of Medicine Ramathibodi Hospital, Mahidol University, Bangkok 10400, Thailand

## Background

Thailand has three main health insurance schemes: Universal Coverage Scheme (UCS), Social Security Scheme (SSS) and Civil Servant Medical Benefit Scheme (CSMBS). Health benefits of these schemes are different, which could lead to inequity in access to care and quality of care. Medical emergency care was the first healthcare programme that was implemented towards harmonization of the three schemes. This programme was implemented since April 1, 2012. Since then, all emergency care patients were expected to be treated under the same rules without being rejected by all private hospitals. The payment mechanism used fee schedule of CSMBS for outpatient service and diagnosis related group (DRG) for inpatient service with a single base rate of 10,500 Baht per adjusted relative weight including medical instruments and drugs. The aim of the study was to assess the policy outcomes of the Emergency Medical Service focusing on private hospitals.

## Materials and methods

Quantitative analysis of emergency claims and in-depth interviews were employed within the present study.

## Results

During the first 6 months of the programme, there were 7,805 patients who accessed 215 private hospitals, 83% of the patients were hospitalised and half of these inpatients were charged from the hospital before receiving medical treatments. Hospitals had been reimbursed for emergency care of approximately 30%-40% of total charge. Ambiguous criteria of emergency conditions led to system abuse of some private hospitals and patients. The utilisation rate of patients under CSMBS was the highest compared to the other two schemes. Hospitalised patients under CSMBS also had a problem about the referral system due to the lack of main contracting hospitals.

## Conclusions

This programme was successful in improving access to emergency care services provided by private hospitals. The standard triage criteria and reimbursement rate contributed to moral hazard from both patient and provider sides. There are needs for setting up the referral system for CSMBS and protection mechanism to prevent the advanced payment.

